# Do patients prefer optimistic or cautious psychiatrists? An experimental study with new and long-term patients

**DOI:** 10.1186/s12888-016-1182-1

**Published:** 2017-01-17

**Authors:** Stefan Priebe, Gonca Ramjaun, Nadia Strappelli, Eleonora Arcidiacono, Eugenio Aguglia, Lauren Greenberg

**Affiliations:** 1 Unit for Social and Community Psychiatry (WHO Collaborating Centre for Mental Health Services Development), Newham Centre for Mental Health, Queen Mary University of London, E13 8SP London, UK; 2 Università degli Studi di Catania, Azienda Ospedaliera-Universitaria “Policlinico-Vittorio Emanuele” Catania, Presidio “Gaspare Rodolico”, U.O.P.I di Psichiatria Via Santa Sofia 78 9s 100, Catania, Italy; 3 Pragmatic Clinical Trials Unit (PCTU) Centre for Primary Care and Public Health, Blizard Institute, Yvonne Carter Building, 58 Turner Street, London, E1 2AB UK

**Keywords:** Psychiatric treatment, Treatment expectations, Pharmacotherapy, Psychological treatment, Communication, Information

## Abstract

**Background:**

Patients seeking treatment may be assumed to prefer a psychiatrist who suggests a new treatment with confidence and optimism. Yet, this might not apply uniformly to all patients. In this study, we tested the hypothesis that new patients prefer psychiatrists who present treatments optimistically, whilst patients with longer-term experience of mental health care may rather prefer more cautious psychiatrists.

**Methods:**

In an experimental study, we produced video-clips of four psychiatrists, each suggesting a pharmacological and a psychological treatment once with optimism and once with caution. 100 ‘new’ patients with less than 3 months experience of mental health care and 100 ‘long-term’ patients with more than one year of experience were shown a random selection of one video-clip from each psychiatrist, always including an optimistic and a cautious suggestion of each treatment. Patients rated their preferences for psychiatrists on Likert type scales. Differences in subgroups with different age (18–40 vs. 41–65 years), gender, school leaving age (≤16 vs. >16 years), and diagnosis (ICD 10 F2 vs. others) were explored.

**Results:**

New patients preferred more optimistic treatment suggestions, whilst there was no preference among long-term patients. The interaction effect between preference for treatment presentations and experience of patients was significant (interaction *p*-value = 0.003). Findings in subgroups were similar.

**Conclusion:**

In line with the hypothesis, psychiatrists should suggest treatments with optimism to patients with little experience of mental health care. However, this rule does not apply to longer-term patients, who may have experienced treatment failures in the past.

**Electronic supplementary material:**

The online version of this article (doi:10.1186/s12888-016-1182-1) contains supplementary material, which is available to authorized users.

## Background

Non-specific factors have been shown to influence treatment outcomes throughout medicine and, in particular, in psychiatry. Treatment expectancy and the quality of the therapeutic relationship are widely seen as central non-specific factors, which apply to both psychopharmacological and psychological treatments [1, 2]. Evidence suggests that patients are more likely to adhere to and benefit from psychopharmacological medication, if their psychiatrists believe in its effectiveness and suggest it with conviction to the patient [3]. In psychological treatments, positive expectations of patients and a helpful therapeutic alliance have been suggested to have a major impact on outcomes across therapeutic schools [4].

Thus, when suggesting a new treatment, psychiatrists need to address patient expectations [5]. Ideally, their explanation as to what to expect from the new treatment should help the patient to trust the psychiatrist and raise the patient’s motivation to start the suggested treatment. So, how can that be achieved? Should psychiatrists be very optimistic about the potential effects of the treatment or may it be sometimes better to be cautious? And if it is the latter, when is a more cautious presentation appropriate?

One may assume that, in general, patients are likely to prefer a psychiatrist who recommends a treatment with confidence and optimism. They have sought the help of an expert to find a remedy for their distress, and should normally prefer a psychiatrist who is convinced that the suggested treatment will be effective rather than a cautious one.

However, this may not apply to all patients. Patients who have been in psychiatric treatment for longer periods of time may have learned that outright optimism about the expected effects of a new treatment is not always justified. Treatments may have failed in the past, or – at least – have not removed the distress for good, otherwise patients would not have to consider another treatment. Thus, such patients may be less convinced by optimism and have more trust in psychiatrists who present new treatments with some reservation about the probable treatment outcomes. In this case, a less optimistic and even explicitly cautious presentation of treatment expectations may instil more trust into both the psychiatrist and the new treatment. In line with some models of clinical communication [6, 7], a very optimistic presentation may raise doubts in these patients and be rather detrimental, whilst a cautious presentation may lead the patient to feel that the psychiatrist is competent and rather expect improvement.

This leads to the hypothesis that patients who are new to psychiatry prefer psychiatrists who suggest new treatments with optimism and conviction, whilst patients with longer experience of psychiatric treatments prefer rather cautious treatment expectations.

We tested this hypothesis in an experimental study, and explored whether the findings would differ in subgroups with different age, gender, school education and clinical diagnosis.

## Methods

The study was a hypothesis testing, non-clinical experiment. Brief video-clips with consultant psychiatrists suggesting treatments in a more optimistic or cautious manner were shown to different patient groups. Patients assessed to what extent they trusted the psychiatrist and wanted to start the new treatment with him or her. The design drew on and further elaborated a method that had successfully been used in a previous study for establishing how psychiatrists should introduce themselves in a first contact [8].

### Psychiatrists

Four Consultant Psychiatrists were purposively selected using age, gender and ethnicity as selection criteria, i.e., two women and two men from different age groups, with one of them being from an ethnic minority. We recruited psychiatrists rather than actors to reflect a real scenario as far as possible, therefore increasing the external validity of the study. All psychiatrists were from different services than the ones in which patients were recruited. This was to avoid any confusion of patients with real scenarios either at the time of the study or later.

### Video-clips

Each psychiatrist was video recorded with four different types of treatment suggestions, i.e., an optimistic and a cautious one, each for medication and psychotherapy.

The format and length of all video-clips was consistent. In the video-clips, only the psychiatrist is seen acting in the introductory phase of a medical consultation with a new patient. Following the technique used in a previous study [8], psychiatrists were asked to talk to an imagined patient sitting behind the camera. The psychiatrists were asked to introduce themselves and suggest a treatment, varying only for the nature of the treatment and the level of optimism or cautiousness. The four presentations were:

- *Optimistic suggestion of pharmacological treatment*
  My name is Dr XX. I am your new consultant psychiatrist. Having looked at your records, I believe we could start you on a new medication, which you will have to take once a day for 6 weeks. I am very optimistic that the new medication will be most effective and make all your symptoms go away.
- *Cautious suggestion of pharmacological treatment*
  My name is Dr XX. I am your new consultant psychiatrist. Having looked at your records, I believe we could start you on a new medication, which you will have to take once a day for 6 weeks. I really cannot say whether it will or will not work with you, and I cannot guarantee any improvement, but it may be worth trying.
- *Optimistic suggestion of psychological treatment*
  My name is Dr XX. I am your new consultant psychiatrist. Having looked at your records, I believe we could start you on psychological therapy. This will be weekly one-to-one sessions with a psychologist for 8 weeks. I am very confident that such talks will be most helpful and sort all your problems.
- *Cautious suggestion of psychological treatment*
  My name is Dr XX. I am your new consultant psychiatrist. Having looked at your records, I believe we could start you on psychological therapy. This will be weekly one-to-one sessions with a psychologist for 8 weeks. I cannot say whether such talks will really help you, and cannot promise anything, but you might want to try.

### Patients

Patients were recruited from secondary services in the East London Boroughs of Newham and Tower Hamlets. We recruited two groups of patients. Overall inclusion criteria for both groups were intentionally wide: current patient in secondary mental health services; 18–65 years of age; a clinical diagnosis of ICD-10 [9] F2, F3 or F4; sufficient command of English to understand the presentations of the psychiatrists and the rating items; and capacity to provide informed consent. The further inclusion criterion was the length of the experience of mental health care. One group had an experience of mental health care of less than 3 months (‘new’ patients), the other group of more than one year (‘long-term’ patients).

Exclusion criteria were; organic mental disorder or learning difficulty; too high a current symptom level to participate in the study.

### Experimental design

A randomised sequence list was generated to ensure that each patient watched four video-clips, one from each psychiatrist, and one from each of the four presentations, i.e., optimistic and cautious presentation of pharmacological and psychological treatment. This design made it possible to eliminate the influence of the psychiatrists from the data analysis.

### Outcome

After watching each clip patients rated their preference on a four item scale. The four items were:

- Do you believe this is a good doctor?
- Would you have trust in this doctor?
- Would you like this doctor to be your psychiatrist?
- Would you like to start the new treatment with this psychiatrist?

Each item was rated on a four-point Likert scale with the categories from 1 to 4: definitely no; probably no; probably yes; definitely yes. The sum score of the four items was taken as the patient’s preference rating of the given video-clips.

### Sample size

The sample size calculation was based on a main effect on the four item preference scale. To compare the average ratings of the two different instructions in the whole sample, with 90% power at the 5% significance level, a total of 190 patients were required to detect a medium effect size of 0.3 (defined as a difference of 1 point between the mean preference ratings for the optimistic and cautious introductions, and assuming a standard deviation of three scale points based on data from a previous study). This assumes that the correlation between each pair of ratings made by each patient is 0.5. To account for the possibility of insufficient data quality in some cases, we decided to recruit 100 patients in each group, thus a total of 200 patients.

### Procedure

Data was collected between May 2014 and December 2015. Patients who were eligible for the study were asked by their psychiatrist whether they would consent to be approached by a researcher. If patients agreed, they were then contacted by a researcher, who explained the study and asked for written informed consent. Consenting patients were shown the video-clips in a quiet room. All interviews were conducted by a trained psychiatrist or psychologist. Patients were reimbursed £10 for their participation. Information on patient’s length of treatment, age, gender, school leaving age, ethnicity and clinical diagnosis were obtained from the patient’s records.

The study was approved by the National Research Ethics Committee London – East (ref. 14/LO/0126).

### Data analysis

The characteristics of the sample were presented using descriptive statistics. The scores of the four video-clips were computed for each patient.

Cronbach’s alpha was calculated to assess the internal consistency of the four scores given by each patient.

Linear mixed effects regression models were used to compare the mean rating of video-clips showing cautious presentations to the mean rating of video-clips showing optimistic presentations. A fixed effect was fitted for psychiatrist. Mean scores were compared for the whole sample. We fitted an interaction between the type of treatment (pharmacological vs. psychological) and type of video-clip (optimistic vs. cautious); the significance of which was assessed using a Wald test. We then fitted an interaction between experience of the patient (new vs. long-term) and the type of video-clip. Moreover, we calculated mean scores and 95% confidence intervals amongst different subgroups of patients. These subgroups included type of treatment, gender, age group (<40 vs. >40 years), diagnostic group (F20-29 vs. other) and school leaving age (<16 vs. >16 years). All statistical analyses were carried out using Stata version 11.0 for Windows [10].

## Results

### Patient characteristics

In total 203 patients were approached by the researchers. Three patients declined participation, and 200 patients were recruited, 137 in out-patient services and 63 in in-patient services. The characteristics of the two groups are shown in Table 1.

**Table 1:** Patient characteristic for long-term and new patients

	New Patients	Long-term Patients
Gender		
Female	40	48
Male	60	52
Ethnicity		
White	49	53
Asian	30	26
Black	16	13
Mixed	2	7
Other	3	1
Diagnosis – ICD 10		
F2	24	22
F3	57	56
F4	19	22
School leaving age		
< 16	56	52
> 16	44	48
Age		
18–40	65	54
41–65	35	46

The socio-demographic and clinical characteristics of the groups were largely similar. In the long-term patients the actual experience of mental health care ranged between two and 46 years.

### Ratings - overall

The 200 patients provided a total of 800 ratings. Internal consistency for the four items was very high with a Cronbach’s alpha of 0.95. Overall, cautious treatment presentations were strongly associated with a lower mean score (i.e., 10.3, SD = 3.6) compared to optimistic presentations (10.9, SD = 3.6) in the whole sample (*P* = 0.007). Psychological treatments (11.0, SD = 3.5) were rated more positively than pharmacological ones (10.2; SD = 3.7; *P* = 0.001). The mean scores for new patients were 10.8 (SD = 3.7) and for long-term patients 10.4 (SD = 3.5; *P* = 0.16). There was no interaction found between the type of treatment, i.e., medication or psychological treatment, and type of clip, i.e., optimistic versus cautious suggestion (interaction *P* = 0.35).

### Ratings for new and long-term patients

The mean difference between optimistic and cautious video-clip scores varied significantly between new and long-term patients (interaction *P* = 0.003). New patients had a lower mean score for cautious video-clips whereas there was no difference in ratings of long-term patients between optimistic and cautious video-clips. Table 2 shows the mean scores and 95% confidence intervals, for new and long-term patients, in subgroups of patients with different gender, age, school-leaving age and diagnosis. There was a larger absolute mean difference for new patients in all subgroups presented.

**Table 2:** Mean scores and 95% confidence intervals of patients’ preference ratings for psychiatrists with optimistic and cautious treatment presentations, separated for new and long-term patients, and each for different subgroups of patients

	New patients			Long-term patients		
		Optimistic suggestion	Cautious suggestion		Optimistic suggestion	Cautious suggestion
	n	Mean (95% CI)	Mean (95% CI)	n	Mean (95% CI)	Mean (95% CI)
Overall*	100	11.5 (11.0, 11.9)	10.1 (9.6, 10.6)	100	10.4 (9.9, 10.9)	10.4 (9.9, 10.9)
Gender						
Female	40	11.1 (10.3, 11.8)	10.4 (9.6, 11.2)	48	9.9 (9.2, 10.7)	10.3 (9.6, 11.1)
Male	60	11.7 (11.1, 12.4)	9.9 (9.2, 10.6)	52	10.8 (10.1, 11.4)	10.5 (9.9, 11.1)
Age group						
18–40	65	11.4 (10.9, 12.0)	9.7 (9.0, 10.3)	54	10.1 (9.5, 10.8)	10.1 (9.4, 10.8)
> 40	35	11.5 (10.5, 12.5)	10.9 (10.0, 11.9)	46	10.7 (9.9, 11.4)	10.8 (10.1, 11.4)
School leaving age						
< 16	29	11.1 (9.9, 12.2)	10.9 (9.8, 12.1)	35	10.5 (9.7, 10.9)	10.9 (10.2, 11.7)
> 16	71	11.6 (11.1, 12.1)	9.7 (9.2, 10.3)	65	10.3 (9.7, 10.9)	10.1 (9.5, 10.7)
Diagnosis (ICD-10)						
F2	24	11.8 (10.8, 12.8)	10.4 (9.2 11.6)	22	11.5 (10.4, 12.6)	9.8 (8.7, 10.9)
Other	76	11.3 (10.8, 11.9)	10.0 (9.4, 10.6)	78	10.1 (9.5, 10.6)	10.6 (10.0, 11.1)

*P-value for interaction = 0.003

*CI* confidence interval, *n* number of patients

## Discussion

### Main findings

The main findings from this experimental study are consistent with the hypothesis. Patients who are relatively new to mental health services prefer psychiatrists with optimistic treatment suggestions, whilst this preference has not been found in patients with longer-term experience of mental health care. The difference between new and long-term patients is highly significant. Thus, the relative preference of each group is in line with the hypothesis. However, the results do not support a further part of the hypothesis, i.e., that long-term patients explicitly prefer more cautious treatment suggestions.

The difference with a higher preference for optimistic presentations in new patients as compared to long-term patients was found in all subgroups. Yet, the study had not been powered to identify significant differences in these subgroups so that the findings in subgroups have to be interpreted as merely descriptive and with caution.

### Strengths and limitations

Most studies on the association between psychiatrists’ communication and patient responses have employed naturalistic designs. Such designs cannot control for various confounding factors in the complex situation of real treatment, capture only the naturalistically occurring variance and do not allow conclusions on causal relationships. Experimental designs are better suited to assess patients’ preferences of different communication styles. The experimental design of this study does allow for the identification of, cause and effect. The presentation of psychiatrists is the cause of patients’ preference, and the design with randomly varied presentations of all video-clips excluded the potential influence of other factors. All psychiatrists were rated with each introduction so that the findings are independent of the socio-demographic characteristics of psychiatrists or their personality. All psychiatrists were unknown to the patients and the findings were not influenced by previous experiences with the psychiatrist. The video-clips were with real psychiatrist to have a maximum of genuineness and credibility. Lastly, there were an equal number of new and long-term patients, and the long-term patients had been in treatment for a minimum of two years so that the difference in experience between the two groups was even more marked than envisaged in the design.

However, the study also has several limitations: As mentioned, it was not sufficiently powered to identify significant interaction effects in subgroups, and to test whether the interaction effect differs for psychopharmacological and psychological treatments. As we focused on the difference between optimistic and cautious presentations, their wording may have been too strong and different from what is likely to happen in routine practice, when more mixed messages are used. Different presentations in between the very optimistic and very cautious ones used in this study may well have led to different patient ratings. It is possible that patients’ preferences are influenced by further factors that were not considered in the study. The study was conducted in secondary mental health services in London, and thus caution must be taken when generalising the results to other populations such as those in primary care and to other regional contexts. Finally, the study assessed the first impression of patients only and did not explore what would happen after a longer talk and attempts to reach shared decisions.

### Implications

There is a general line in medicine that more optimistic physicians achieve better treatment outcomes. This is underpinned by some evidence in general practice, in the treatment of cancer patients, and pharmacological interventions [11–13]. This is also consistent with the concept of hope in psychiatry [14]. In accordance with this general line, the findings of this study suggest a clear guideline for psychiatrists when they recommend new treatments to patients who are relatively new to mental health care and have not yet had long term experiences with other psychiatrists and their treatments. To these patients, psychiatrist should present the suggested treatment option with confidence and optimism in order to establish trust and strengthen the patient’s motivation to start the new treatment. When talking to patients with longer experience of psychiatric treatments, they might want to be more cautious, and optimism does not necessarily lead to more positive patient responses. These patients might still benefit from more optimism and hope. If so, decidedly optimistic psychiatrists seem not to trigger such optimism in the patients, perhaps because the confident announcement of reliably positive effects lacks credibility. Yet, there is no evidence that psychiatrists should be particularly cautious with these patients either. Thus, psychiatrists need to decide patient by patient as to how best to present the treatment they think is appropriate. It remains unclear which characteristics of the patient or the given context should guide their decision, as the characteristics assessed in this study did not seem to make a major difference. Future research may explore whether other forms of presentations, possibly with explicit reference to disappointing experiences in the past, lead to more trust and more positive expectations in patients with longer term experience of care.

## Conclusions

How psychiatrists present treatment expectations makes a difference to the patient’s attitude towards the psychiatrist and the suggested treatment. More optimistic presentations are preferable with new patients, but not necessarily with those who have already been in treatment for more than a year.

Much of good communication may be determined by personal style and complex skills [15]. Yet, some aspects may also be based on empirical evidence. Such evidence is difficult, if not impossible, to provide in naturalistic studies. For future research, more experimental studies should aim to address practical questions of how psychiatrists should communicate and provide evidence that is very relevant for everyday routine practice.

## Additional file

Additional file 1: Data set video clips BMC Psychiatry spreadsheet. (XLS 168 kb)

## Abbreviations

ICD-10: International Classification of Diseases and Related Health Problems 10^th^ revision
